# A rare case report of heavy dose colchicine induced acute kidney injury

**DOI:** 10.1186/s40360-018-0260-z

**Published:** 2018-10-30

**Authors:** Hongzhen Zhong, Zhiqing Zhong, Hongyan Li, Tianbiao Zhou, Weiji Xie

**Affiliations:** 1 Department of Nephrology, the Second Affiliated Hospital of Shantou University Medical College, Shantou, 515041 China; 2 Department of Nephrology, Huadu District People’s Hospital of Guangzhou, Southern Medical University, Guangzhou, China

**Keywords:** Colchicine, Acute colchicine intoxication, Acute kidney injury, Continuous renal replacement therapy (CRRT), Treatment

## Abstract

**Background:**

Colchicine is a natural alkaloid that is mainly used for the treatment of inflammatory diseases. Effective and toxic doses are very similar, but case reports of higher colchicine doses inducing acute toxicosis is rare.

**Case presentation:**

A 19-year-old woman was sent to the emergency room for taking 80 colchicine tablets (0.5 mg per tablet) 44 h previously. The main physical symptom was abdominal pain. Following ingestion, the patient suffered multi-system failure including renal, respiratory, circulatory, and digestive. Continuous renal replacement therapy (CRRT) and other treatment measures were used to remove metabolic wastes and poisons, and to treat other complications. Renal function was restored after a series of treatments.

**Conclusion:**

We report a case of an acute kidney injury induced by an overdose of colchicine. CRRT and a series of related treatments were beneficial for the treatment of colchicine poisoning.

**Electronic supplementary material:**

The online version of this article (10.1186/s40360-018-0260-z) contains supplementary material, which is available to authorized users.

## Background

Colchicine is a natural alkaloid that is mainly used for the treatment of inflammatory diseases, such as gout and familial Mediterranean fever. Poisoning, a major public health concern around the world, is a frequent cause of referral to medical emergencies, and requires a rapid and precise diagnosis for adequate treatment [1, 2]. Effective and toxic doses are very similar, but case reports of higher colchicine doses inducing acute toxicosis is rare [3]. Colchicine intoxication is often accompanied by severe adverse complications and mortality, and there is no antidote, so it represents a clinical toxicology emergency [4]. We report a case of an acute kidney injury induced by a high dose of colchicine to as a clinical example for the treatment of acute colchicine intoxication.

### Case presentation

On 2018.01.24, a 19-year-old woman was admitted to the emergency room after taking 80 colchicine tablets (0.5 mg per tablet) 44 h previously. She had an argument with her boyfriend and ingested the colchicine to commit suicide. She was previously healthy and had no history of drug allergies. The clinical symptoms were abdominal pain, watery diarrhea and profuse vomiting. Other symptoms were muscle weakness and palpitations.

On physical examination, the temperature was 38.7 °C, pulse rate was 145, and respiration rate was 39. Her blood pressure was 122/60 mmHg, and she weighed 43 kg. Physical examination indicated upper abdominal pain.

Laboratory test results before treatment indicated the following: a white blood cell (WBC) count of 28.2 × 109/L, and other values such as red blood cell (RBC) count, hemoglobin (HGB) level and platelet (PLT) count were within the normal ranges. The levels of α-L-fructosidase (AFU), adenosine deaminase (ADA), alanine aminotransferase (ALT), aspartate aminotransferase (AST), alkaline phosphatase (ALP) and lactate dehydrogenase (LDH) were increased to 98, 57, 84, 408, 378 and 3494 respectively from reference values (reference range were 12–40 U/L, 0–50 U/L, 5–40 U/L, 8–40 U/L, 40–150 U/L, 109.0–245.0 U/L, respectively). Biochemical abnormalities also included hypokalemia and hypoglycemia. Plasma prothrombin time (PT) and activate part plasma prothrombin time (APTT) were significantly prolonged at 23.50 s and 52.40 s respectively. The level of N-terminal pronatriuretic peptide (NT-proBNP) was 5950 pg/mL, which is abnormal with values higher than 450 pg/mL in the populations under 50-year-old (referrence value). The Electrocardiograms revealed sinus tachycardia.

Hemoperfusion was performed to remove circulating toxins. The patients refused other treatments in Department of Emergency. After 44 h later, gastrointestinal hemorrhage, acute liver injury, acute kidney injury and acute cardiac damage were reported, along with prolonged coagulation. She was then admitted to the intensive care unit. Adequate fluid and electrolyte replacement, oxygenation and other supportive cares was initiated. Anti-inflammatory ceftriaxone sodium was used. Since the unobstructed drainage tube revealed brown fluid, gastric lavage and charcoal were not recommended.

During two days after admission, she presented with high fever, subcutaneous hemorrhage and anuria. Arterial blood gas analysis suggested hyperlactinemia. Uric convention and occult stool were positive for blood. The level of Creatine Kinase-MB had sharply increased to 182 U/L and HGB level and PLT count rapidly plunged to 49 g/L and 11 × 10^9^ /L, respectively. APTT had increased to 72.4 s. At that time, renal function deteriorated and anuria was observed, and the levels of serum creatinine (Cr) and blood urea urea (BUN) were elevated. CRRT was used to remove metabolic wastes and poisons and promoted recovery of renal function. RBC, PLT and plasma were transfused to alleviate anemia and deficient coagulation.

Due to the high dosage of colchicine ingestion, the patient progressed to exhibit shortness of breath, high fever, and subsequent coma. The vital signs at this point were: a heart rate of 87, respiration rate of 21, and the blood pressure was 103/52 mmHg. Blood lactic acid levels were slightly increased. Hyperleukocytosis, low hemoglobin and thrombocytopenia were present. Emergency treatment with tracheal intubation via oral cavity was initiated.

On 2018-02-01, the level of BUN and CK were elevated and anuria was noted. Maintenance therapy with CRRT was initiated to clear metabolic toxins. The arterial blood gas results were generally normal, and she was extubated and provide with supplemental oxygen through a nasal tube. The WBC count returned normal levels, and blood coagulation and liver function were gradually normalized as well.

Following a month of treatment, urine volume increased, but renal function indicators remained abnormal. In addition, HGB levels gradually increased but remained low. Intermittent CRRT and diuretics were administered.

**Fig. 1:**
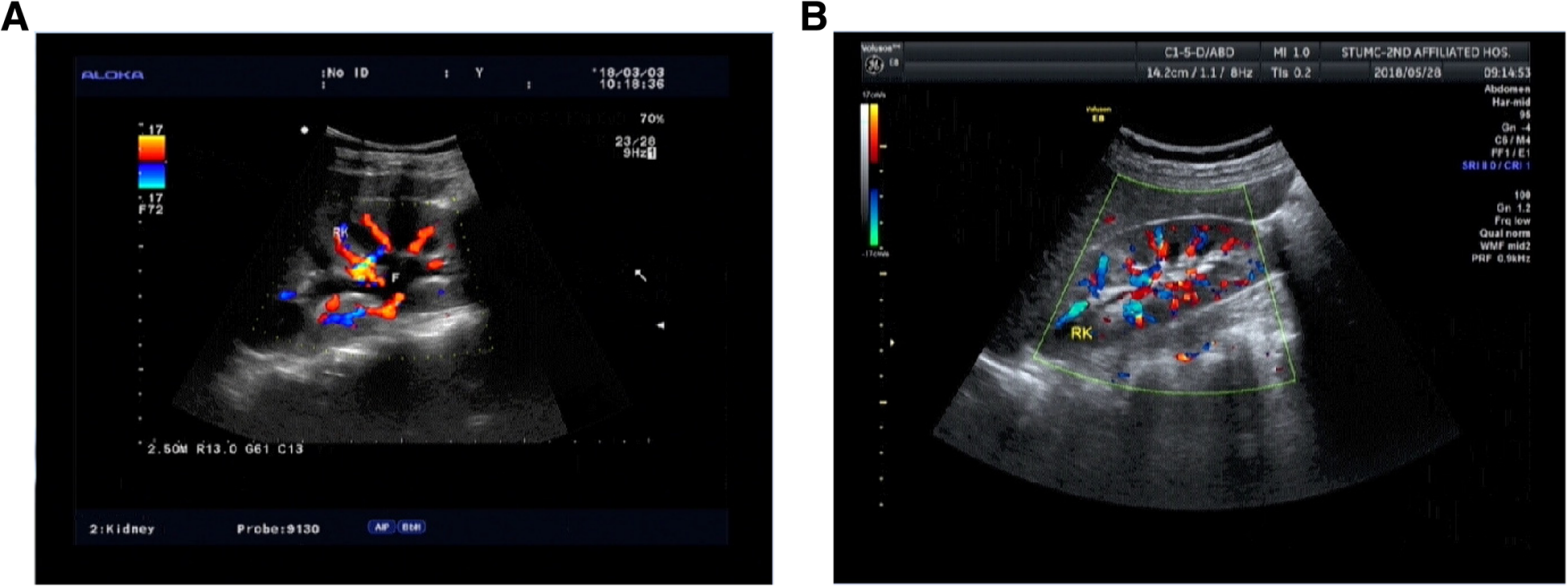
Renal ultrasound indicated: **a**: Pre-treatment situation: The kidneys have clear outlines, normal shapes, smooth capsules, increased cortical echo, reduced medulla echo, and clear boundaries of the cortex and medulla. b: After treatment: The kidneys have clear contours, normal size and shape, smooth capsules, clear boundaries between the medulla and cortex, and even echoes

**Fig. 2:**
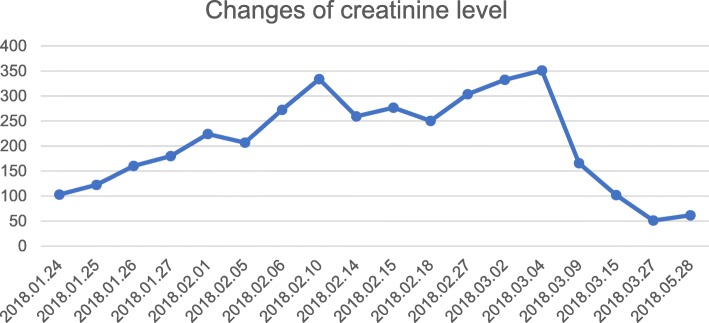
3 days after colchicine ingestion, the level of creatinine increased gradually. CRRT was performed since admission, and creatinine level mildly increased. After an intermittent hemodialysis, the level of creatinine dropped off significantly, and then it returned back to normal level

**Fig. 3:**
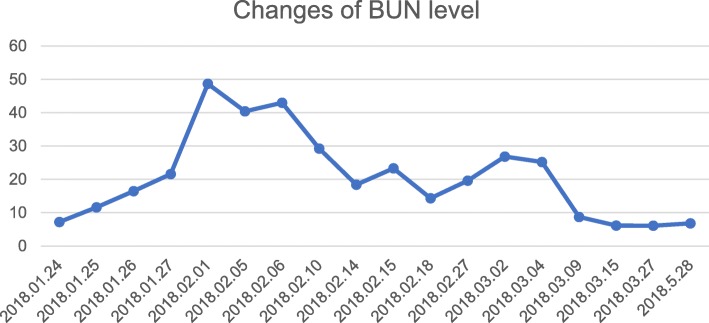
The level of BUN increased to the pole 10 days after colchicine ingested. CRRT was performed to remove metabolite. As other indicators were generally normal, intermittent hemodialysis was administered. BUN gradually returned to normal

**Fig. 4:**
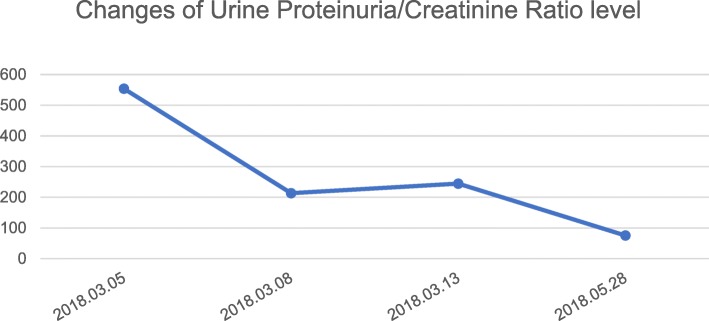
Following treatment, the urinary proteinuria/creatinine ratio decreased significantly, and then returned to normal

On 2018-03-06, her urine volume reached 3000 ml. Renal ultrasound showed the kidneys were full and diffusely changed (Fig. 1a). Urinary albumin-to-creatinine ratio (reference range of 0–200 mg/g) was also abnormal as displayed in Fig. 4. Intermittent hemodialysis was performed and renal function improved. Three days later, the levels of Cr and BUN were also normal, and the patient was discharged. (Blood and urine specimens, and blood biochemistry were reviewed one week later. These values and the time of CRRT treatment are shown in Table 1. Changes of Cr level and BUN level are presented in Figs. 2 and 3. On 2018.05.28, the renal ultrasound urinary albumin-to-creatinine ratio were normal (Fig. 1b and 4). The treatment timeline is shown in Additional file 1: Figure S1.

**Table 1:** Laboratory test and time of CRRT treatment

Date	Time of CRRT treatment(hour)	total Ultrafiltration volume(mL)	urine volume (mL)	Lac (0.5–1.8)mmol/L	Cr (44.0–133.0)μmol/L	BUN (2.90–8.20)mmol/L	electrolyte imbalance	WBC (3.5–9.5) × 10^9/L	RBC (3.80–5.10) × 10^12/L	HGB (115–150)g/L	PLT (100–300)10^9/L
2018.01.26③	31.5	8820	< 100	2.9	160.2	16.46	no	3.7	1.63	49	11
2018.01.27	25	3920	< 100	1.4	179.9	21.59	no	2.5	2.3	67	10
2018.01.29④	28	6000	< 100	2.5	*①	*	no	9.6	2.43	73	11
2018.01.31	22	7770	< 100	2	*	*	no	22.4	2.75	81	8
2018.02.01	16	5680	< 100	2.1	224	48.67⑤	no	34.4	2.3	68	7
2018.02.03	29.5	8160	< 100	1.3	*	*	no	35.3	1.97	59	8
2018.02.04	16.5	5270	< 100	*	*	*	no	*	*	*	*
2018.02.05	8.5	4050	< 100	1.3	206.8	40.41	no	42	2.2	68	59
2018.02.06⑥	10	3630	< 100	0.9	272.4	42.97	hypocalcemia		*	*	*
2018.02.08	8.5	1890	< 100	1.6	*	*	hypocalcemia	31.2	1.95	62	88
2018.02.09	4	690	< 100	1.1	*	*	hypocalcemia	37.9	1.32	44	76
2018.02.10	23	8700	< 100	1.2	*	*	hypocalcemia	46.4	2.16	97	101
2018.02.14	16.5	6900	< 100	0.9	259.3	18.4	hypocalcemia	11.4	1.75	59	152
2018.02.15	16	4580	< 100	0.8	276.7	23.3	no	*	*	*	*
2018.02.18⑦	12.5	6100	< 100	0.6	250.3	14.33	no	8.2	1.61	55	83
2018.02.21⑧	14	6800	< 100	0.8	*	*	no	5.8	1.63	56	88
2018.02.23	7.5	3360	< 100	*	*	*	no	*	*	*	*
2018.02.26	14	4470	< 400	1.1	303.6	19.6	no	6.5	1.17	48	352
2018.03.02	*	*	1000	*	332.5	26.83	no	7.2	2	71	445
2018.03.04	*	*	3100	*	351.1⑨	25.2	no	7.5	2.18	75	381.5
2018.03.09	*	*	1800②	*	165.7	8.72	no	7.7	2.36	79	324
2018.03.15	*	*	normal	*	102	6.14	no	10.35	2.88	88	308
2018.03.27	*	*	normal	*	51.1	6.1	no	9.4	3.36	98	281
2018.05.28	*	*	normal	*	61.6	6.8	no	7.5	4.12	118	213

Note:① *: That item was not administered that day. ②Diuretics were discontinued.③Pancytopenia and coagulation abnormalities were present.④Liver function test showed: AST 3283u/l, ALT 813 u/l; AFU103u/l. Besides, the patient developed shortness of breath and comatose. Mechanical ventilation was then performed. ⑤The BUN level and PLT count were life-threatening. ⑥FIB 0.56 g/L, D-Dimer 25.26μg/ml with no active bleeding; one possible cause was colchicine intoxication. APTT> 200 s, PT17.5 s, FDP55.2μg/ml; the abnormal values might be associated with a stress reaction. ⑦Mechanical assistance was removed. ⑧Coagulation abnormalities were resolved. ⑨Intermittent hemodialysis was initiated

## Discussion and conclusions

Colchicine has a low therapeutic index and ingestion of a dose of 0.8 mg/kg can result in death [5, 6]. The total quantity of colchicine taken in this case was 40 mg, and his body weight was 43 kg. Colchicine poisoning is an uncommon but life-threatening, and there is no effective antidote at present. Supportive therapy is the primary option [7–9]: (1) gastric lavage within 60 min of ingestion, following by activated charcoal help prevent enterohepatic recirculation of the drug. (2) Early fluid resuscitation and multi-organ adjuvant therapy should be performed as soon as possible. (3) Colchicine is a neutral fat-soluble alkaloid, and it reaches the peak serum concentration after 0.5–3.0 h. Hemodialysis and hemoperfusion are ineffective, and plasma exchange and CRRT can be applied if necessary. (4) Symptomatic supportive treatment for complications, such as pulmonary infection, hematopoietic suppression, should be initiated.

Colchicine is a lipophilic compound and is rapidly absorbed from the gastrointestinal tract after oral administration. It’s plasma peak concentrations are detected at 0.5 to 3 h after ingestion [10]. After assessment of 17 pediatric cases with colchicine poisoning, Alaygut et al. [11] concluded that patients arrived at the hospital on average 7.3 h after taking colchicine. We reviewed the medical history of this case and found that the patient was sent to hospital 6 h after ingesting colchicine. However, the patient refused all treatment in emergency department. Forty-four hours after being admitted, gastrointestinal hemorrhage, acute liver injury, acute kidney injury and acute cardiac damage were reported, along with deficient coagulation. Based on these observations, gastric lavage and activated charcoal were not administered.

Various factors such as depletion of volume/hypotension, rhabdomyolysis and multi-organ failure contribute to renal failure after colchicine intoxication [12]. Up to 20% of colchicine is excreted by the kidneys [13, 14], and this is often associated with renal impairment [15]. In the current case, depletion of volume/hypotension and rhabdomyolysis was likely to be the primary cause of acute renal insufficiency and continuous anuria. In addition, cells with a high division index, such as gastrointestinal epithelial cells, renal tubular epithelial cells (RTEC), and bone marrow hematopoietic cells are highly susceptible to the toxic effect of colchicine [16]. Therefore, RTEC toxicity to colchicine was also a critical cause of acute kidney injury and continuous anuria. Fab fragments is reported to be of value in the treatment of colchicine poisoning in animal studies [6, 17–19] and clinical application [20], but it is not currently available and there are few clinical reports. There, supportive therapy was chosen for the patient. Fluid and electrolyte supplementation and CRRT were performed to improve renal perfusion/hemodynamics and address impaired kidney function respectively. As the volume of urine increased, the intermittent hemodialysis was performed to eliminate uremic toxins instead of CRRT. Renal function gradually recovered with this treatment regimen.

There are some limitations to this case study. Mostly notably a lack of colchicine concentration monitoring during the clinical treatment. The absence of renal biopsy is also unfortunate, but the diagnosis was clear based on the presented values. Lastly, the exact mechanism of colchicine poisoning remains unknown, and there is no effective antidote.

In conclusion, we present a case of acute kidney injury and severe complications induced by high dose colchicine following delayed treatment. We report that applied CRRT in the oliguria stage was beneficial for treatment, particularly with alleviating the progression of renal damage. Renal function and morphology returned to normal four months after the initial presentation.

## Additional file

Additional file 1: **Figure S1.** Treatment timeline of this patient. (DOCX 134 kb)

## Abbreviations

ADA: Adenosine deaminas
AFU: α-fucosidas
ALP: Alkaline phosphatase
ALT: Alanine aminotransferase
APTT: Activate part plasma prothrombin time
AST: Aspartate aminotransferase
BUN: Blood urea urea
Cr: Serum creatinine
CRRT: Continuous renal replacement therapy
HGB: Hemoglobin
LDH: Lactate dehydrogenase
NT-proBNP: N-terminal pronatriuretic peptide
PLT: Platelet
PT: Plasma prothrombin time
RBC: Red blood cell
RTEC: Renal tubular epithelial cells
WBC: White blood cell
