# Supplementary figures and images for: A rare case report of heavy dose colchicine induced acute kidney injury
Source: BMC Pharmacol Toxicol. 2018 Oct 30;19:69. doi: 10.1186/s40360-018-0260-z (PMC6208074; doi:10.1186/s40360-018-0260-z)

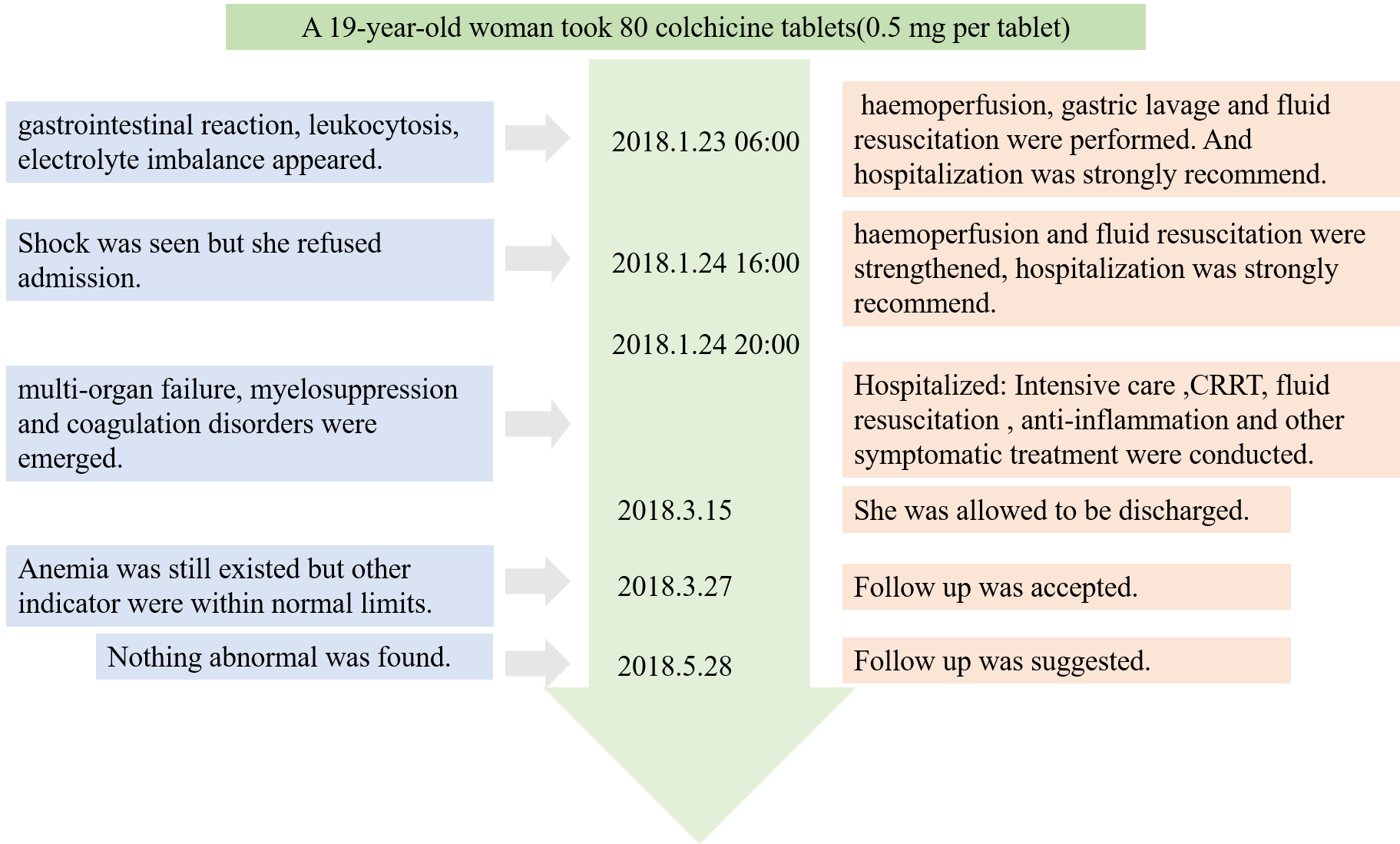


Additional Figure 1.

Supplement: Supplementary file 1 — Figure S1. Treatment timeline of this patient. (DOCX 134 kb) [file 40360_2018_260_MOESM1_ESM.docx]
